# Assessing the Feasibility and Efficacy of Virtual Reality Navigational Training for Older Adults

**DOI:** 10.1093/geroni/igae099

**Published:** 2024-12-12

**Authors:** Tong Bill Xu, Armin Mostafavi, Walter R Boot, Sara Czaja, Saleh Kalantari

**Affiliations:** Human Centered Design Department, Cornell University, Ithaca, New York, USA; Human Centered Design Department, Cornell University, Ithaca, New York, USA; Division of Geriatrics and Palliative Medicine, Weill Cornell Medicine, New York, New York, USA; Division of Geriatrics and Palliative Medicine, Weill Cornell Medicine, New York, New York, USA; Human Centered Design Department, Cornell University, Ithaca, New York, USA

**Keywords:** Spatial learning, Spatial navigation, Spatial presence, User experience

## Abstract

**Background and Objectives:**

This study evaluates the feasibility of virtual reality (VR) wayfinding training with aging adults and assesses the impact of the training on wayfinding performance.

**Research Design and Methods:**

49 participants were recruited using a convenience sample approach. Wayfinding tasks were conducted by 3 participant groups: active VR training, passive video training, and no training, assigned randomly. The training featured 5 tasks in a digital version of a real building. Post-training assessments used 10 tasks in this same building, half of the tasks familiar from the training and half new. The study was double-blinded, with each intervention lasting 10 min. The primary outcomes include the Distance Traveled and Duration for each wayfinding task, with a fixed 10-min limit.

**Results:**

Participants in the VR group reported moderate usability and a high sense of Self Location in the environment with respect to the training intervention. No significant differences were found in performance for the first group of similar wayfinding tasks; however, in the subsequent set of new tasks the VR group significantly outperformed the Control group. This suggests a possible spatial learning effect across multiple exposures (VR training followed by similar task). No adverse effects were reported during or post intervention.

**Discussion and Implications:**

This study provides preliminary evidence that VR training can help to improve wayfinding performance in older adults with no reported adverse effects.

**Translational Significance:** This study addresses the challenge of declining wayfinding abilities in aging adults, a critical issue that can negatively affect quality-of-life. By evaluating the feasibility and effectiveness of virtual reality (VR) wayfinding training, we demonstrate preliminary evidence that VR can improve wayfinding performance in older adults without adverse effects. Implications for translation include the potential to improve individual autonomy and safety, reduce the risk of getting lost, and ultimately enhance the quality-of-life for aging populations by incorporating VR training into routine care or rehabilitation programs.

The United States is expected to see a considerable shift in its age demographics by 2030, when over 70 million individuals, or roughly one-fifth of the total population, will be over the age of 65 (Vespa et al., 2020). Health concerns linked to older ages, such as mild cognitive impairment (MCI), will continue to increase in prevalence due to this demographic shift. Current data suggest that the incidence of MCI could climb from its current level of about 22 per 1,000 individuals to well over 60 per 1,000 individuals (Gillis et al., 2019). Research has shown that older adults tend to underperform in wayfinding and route-learning tasks and struggle with reading signage and acquiring configural knowledge when compared to their younger counterparts (Aubrey et al., 1994; Head & Isom, 2010), and that these wayfinding performance challenges are exacerbated among those with MCI, or Alzheimer’s disease and related dementias (Davis et al., 2017). The declines in wayfinding abilities among older adults can negatively affect their overall quality-of-life (Taillade et al., 2013).

Digital games have been suggested as a promising avenue for mitigating some of these age-related deficits and helping older adults maintain their navigational skills. Prior studies have found that computer-based training or games may enhance cognitive functions associated with wayfinding (Green & Bavelier, 2003; Law et al., 2022), and possibly motor skills (Castel et al., 2005). In a more general sense, studies suggest that game-like interventions can enhance cognitive functions or manage cognitive impairment in older adults (Kelly et al., 2014) and promote general learning (Green & Bavelier, 2012). However, findings regarding transfer of learning, the ability to apply skills learned in a gaming or training context to real-world scenarios (Barnett & Ceci, 2002), have been limited in studies of “brain training” interventions (Owen et al., 2010).

Virtual reality (VR) can simulate complex real-world environments in a controlled setting. Research has shown that VR trainings improve cognitive functions (Anguera et al., 2013; Wais et al., 2021), physical functions (Molina et al., 2014) in older adults, as well as spatial learning (Meade et al., 2019; Parong et al., 2020). Further, research has shown that 3D models are more helpful than 2D floorplans for wayfinding (Verghote et al., 2019) and suggests that VR could have an advantage over desktop programs in terms of transferring navigational skills to the real world (Hejtmanek et al., 2020).

In this study, we designed and developed a VR wayfinding training platform specifically for older adults, with realistic, task-based simulations (rather than artificial training exercises), to improve wayfinding skills. The research aim was to evaluate the feasibility and impacts of the VR training, including user-experience metrics, effects on wayfinding performance, and spatial learning outcomes.

## Method

### Experiment Design

The study was a randomized design with the intervention training condition (VR, Video, Control) as the between study variable. The participants were assigned randomly into one of three intervention groups (VR, Video, or Control, 1:1:1) according to a randomization list generated in the R language.

The study was double-blinded; neither the experimenters nor the participants were aware of the exact purpose of the study or its hypotheses. The experimenters were, however, aware of the difference in interventions in order for them to be able to conduct the study.

### Apparatus

The virtual interior environment that the researchers created for navigational training was based on Martha Van Rensselaer Hall at Cornell University. Most of the modeling and UV mapping for this environment was conducted using Autodesk 3ds Max. Texturing, lighting, and interactivity were added using the Unreal Engine 4.27. All of the front-end interaction and user interactivity leveraged the Blueprint platform and C++ scripting. The VR environment, as well as the videos watched by the Video and Control groups, were run on a Dell Alienware computer equipped with Nvidia RTX 2080 Ti to minimize the risk of latency. The VR environment was presented to participants using a cable-connected Meta Quest 2 head-mounted display at a resolution of 1832 × 1920 pixels per eye (90 Hz, FOV 90°). The video for the control participants in the Video and Control groups was seated in the same position while observing the relevant content on a 24-inch monitor (1920 × 1080 resolution, 60 Hz).

**Figure 1.**
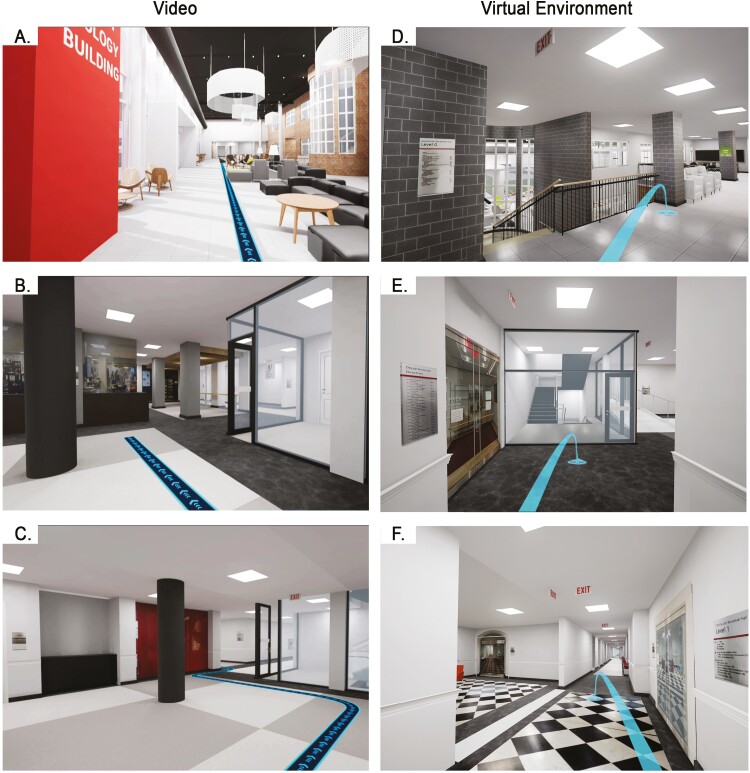
Screenshots from the VR and Video conditions. Images **A–C** show the 2D video, which included blue lines on the floor to guide participants’ attention to the route. The camera in the videos moved inevitably along these predefined routes to the task destinations. Images **D–F** show 2D captures from the immersive 3D virtual environment. The light blue lines shown in these images are movement trajectories actively defined by participants as they sought to complete wayfinding tasks.

Participants experienced the VR environment from a seated position at a desk and were able to look around freely to see various aspects of their virtual surroundings while using the controller to move (teleport) through the environment (Figure 1). They started tasks at the start location and were asked to find the end location by a prompt in VR, with guidance lines on the floor (Figure 1D–F). Videos for the Video group were rendered in Unreal with the camera moving along the shortest path from the start location to the end location (Figure 1A–C). Video used for the Control group was a TED talk “Designing for virtual reality and the impact on education” by Alex Faaborg (TEDx Talks, 2015).

### Participant Recruitment

A convenience sampling approach was used to recruit the older adult participants residing in Ithaca, New York, United States. This involved the distribution of fliers and posters in local retirement communities and sending e-mails to senior community mailing lists. To be eligible, individuals needed to be aged 58 or above and unfamiliar with the building used for wayfinding tasks in the experiment. Participants were excluded if they had substantial mobility issues, visual impairments, a history of epilepsy or motion sickness, medical implants, or scored below the recommended dementia threshold of 19 on the Montreal Cognitive Assessment (Milani et al., 2018).

### Outcomes and Measures

The details of all measurement tools are summarized in Table 1. The baseline measurements included Sense of Direction, Cognitive Assessment, Computer Proficiency, and Mobile Device Proficiency. After the intervention we collected three feasibility measures: Perceived Spatial Presence (Self Location and Possible Action), System Usability, and Motion Sickness. The primary outcome variable was wayfinding performance, operationalized as Time Spent and Distance Traveled on each wayfinding task with a fixed 10-min limit. The researchers developed a Python-based application to track participant trajectories through the real-world building over time, which was used to measure the Distance Traveled (Wayne et al., 2024). Secondary outcomes at the task level were wayfinding experience, including Spatial Anxiety and Workload, and spatial learning, evaluated via two commonly used metrics: after completing each of the real-world wayfinding tasks, participants were asked to point in the straight-line direction where they believed the task’s starting point was located (Pointing Error) and to estimate the straight-line distance to that origin point (Distance Error).

**Table 1.** Baseline and Outcome Measures

Construct	Measures	Reference	Theoretical range	Time of measurement
*Baseline measures*				
Sense of Direction	Santa Barbara Sense of Direction (SBSOD)	Hegarty et al. (2002)	[1, 7]	Online demographic survey
Cognitive Assessment	Montreal Cognitive Assessment (MoCA)	Nasreddine et al. (2005)	[0, 30]	In lab, before experiment
Computer Proficiency	Computer Proficiency Questionnaire (CPQ-12)	Boot et al. (2015)	[6, 30]^a^	Online demographic survey
Mobile Device Proficiency	Mobile Device Proficiency Questionnaire (MDPQ-16)	Roque and Boot (2018)	[8, 40]^a^	Online demographic survey
*Feasibility measures*				
Spatial Presence	Self Location (SPSL): 4-item 5-point Likert Scale	Vorderer et al. (2004)	[1, 5]	After intervention
	Possible Actions (SPPA) 4-item 5-point Likert Scale	Vorderer et al. (2004)	[1, 5]	After intervention
User Experience	System Usability (SUS): 10-item 5-point Likert Scale	Brooke (1996)	[0, 100]^a^	After intervention
	Motion sickness: 16-item 4-point Likert survey	Kennedy et al. (1993)	[0.00, 235.62]^a^	After intervention
*Main outcomes*				
Wayfinding Performance	Task Duration	Ruddle and Lessels (2006)	[0, 600]^b^	Timed by experimenters
	Distance Traveled	Ruddle and Lessels (2006)	[0, ∞]	From recorded trajectory
*Secondary outcomes*				
Wayfinding Experience	Spatial Anxiety: 5-item 4-point scale	Zsido et al. (2020)	[1, 4]	After each task
	Workload: 6-item 7-point Likert Scale	Hart and Staveland (2019)	[1, 7]	After each task
Spatial Learning	Pointing to Task Origin (Pointing Error)	Schinazi et al. (2013)	[0, 180]^c^	After each task
	Distance Estimation from Task Origin (Distance Error)	Schinazi et al. (2013)	[0.0, 4.6]^d^	After each task

^a^Rescaled to theoretical ranges per instruments’ instruction.

^b^The maximum time limit for wayfinding tasks was set to 600 s.

^c^Pointing error was measured in degrees difference from the actual direction.

^d^For the accuracy of Distance Estimation we used the absolute log value of the ratio (participants’ estimates/correct distance); the cap of 4.6 is a 100-times difference.

### Procedure

The study took place in Ithaca, New York, United States from January 2023 to June 2023. Prior to any research activities, the study protocol was evaluated and approved by the Institutional Review Board at Cornell University and informed written consent was obtained from all participants. Experiment sessions were held for one participant at a time and took place in Martha Van Rensselaer Hall. Participants were asked to complete a demographic survey online before attending their experiment session; then upon their arrival they completed the Montreal Cognitive Assessment test. A de-identified dataset and materials related to this study are available at OSF (https://osf.io/63t5z/). This repository has been established in line with ethical and legal guidelines to facilitate further research while ensuring participant confidentiality and privacy.

To ensure similarity between activities prior to wayfinding tasks across conditions, all participants completed a training session about teleportation interaction in VR without wayfinding element with headset on before the intervention. Next, the participants engaged in the VR training which involved viewing five real-world wayfinding tasks in the VR group, watching a wayfinding video covering the same tasks in the Video group, or watching an unrelated video in the Control group (see Apparatus for details). The duration of the training session was 10 min across all conditions. Participants were not informed about the assessment tasks during training. After completing the training, participants were asked to complete the feasibility measures (see spatial presence and user experience in Table 1).

The participants then completed two sets of wayfinding activities, each containing five tasks. In each task, they started from one location and were asked to find another location in the building. The first five real-world tasks (*similar tasks*) had the same start and end locations of the wayfinding tasks included in the VR/Video training sessions. The second five real-world tasks (*new tasks*) took place in a different part of the building that was not included in training.

Each set of wayfinding tasks formed a loop (Supplementary Table 1, Supplementary Figure 2), and each participant was assigned to a random starting point within the loop. For example, one participant might start with Task 3 and from there complete, in order, Tasks 4, 5, 1, and 2. Each wayfinding task was designed to take approximately 3–6 min to complete; if a participant did not finish a task within 10 min then the researchers stopped the data collection for that task and led the participant to the destination. This cutoff minimized the risk of informative right censoring (Shih, 2002; Templeton et al., 2020) and participant fatigue. A procedure flowchart was provided in Supplementary Figure 1.

### Statistical Analysis

Following the Consolidated Standards of Reporting Trials (CONSORT) 2010 recommendations for multi-arm trials (Juszczak et al., 2019), we directly compared the VR group versus the Control group, and the Video group versus the Control group, rather than using a multivariate model with adjustments for multiple comparisons. This analysis took the form of *t*-tests at the 0.05 significance level. We used G*Power software to calculate the required sample size, taking into account potential data clustering (i.e., each participant completed five trials) with an assumed intra-cluster correlation of 0.05; with an assumed medium effect size of *d* = 0.50 and one participant dropout/exclusion per condition. This analysis indicated that our sample size of 14 participants per condition would reach at least 0.80 statistical power.

The data were analyzed at the task level using the R statistical programming language (R Core Team, 2023). Data from Tasks 1 to 5 (similar tasks) and from Tasks 6 to 10 (new tasks) were analyzed separately. For the primary wayfinding performance outcomes (Duration, Distance), we fitted mixed-effect Cox regression models (library “coxme”) with fixed effects of intervention condition, adjusted for the fixed effects of task order, Sense of Direction, and the random effects (intercept) of participant and task. For secondary outcomes, we fitted linear mixed models with the same predictors. We then used the library “emmeans” to estimate the hazard ratio (for primary outcomes) and mean differences (for secondary outcomes), along with 95% confidence intervals (CIs). We controlled for Sense of Direction due to its direct connection to wayfinding ability (Hegarty et al., 2002).

It is important to note that the analysis presented previously is relevant to the task/trial-level outcomes. The measurements of user experience collected immediately after the interventions (i.e., sense of Spatial Presence during the intervention, System Usability, and Motion Sickness) were analyzed at the participant level, and thus have weaker statistical power. These user experience evaluations may be prone to type II error which is a potential failure to observe actually existing differences between the conditions. These specific measures of user experience are therefore treated only observationally in the data analysis (“Feasibility Measures” section).

## Results

### Participants

We recruited a total of 49 participants from January 2023 to June 2023. Five participants were asked to leave before finishing all of the tasks: two participants in VR group, one in Video group, and one in Control group did not finish the last five tasks, one in Video did not finish the last three tasks. Two due to their schedules and three without giving specific reasons. In addition, we excluded 28 invalid trajectories due to incorrect start/end points, result in missing data in Distance measure (Table 3). No participant reported any adverse effects during training or the data collection session.

**Table 3.** Effects of VR Training and Video Training on Primary Outcomes

Measures	VR^a^	Video^a^	Control^a^	VR vs Control^b^	*z*	*p* Value	Video vs Control^b^	*z*	*p* Value
*Similar Tasks*									
*N*	85	80	80						
Duration	262.67 (181.00)	241.90 (169.98)	235.16 (174.72)	0.68 [0.44, 1.05]	−1.74	.081	0.77 [0.49, 1.20]	−1.15	.249
Distance	174.69 (121.13)	189.81 (135.22)	177.99 (146.80)	0.84 [0.58, 1.21]	−0.95	.342	**0.68** **[0.46, 1.00]**	**−1.97**	**.049**
Excluded^c^	5 (5.9%)	4 (5.0%)	3 (3.8%)						
Finished	72 (85%)	73 (91%)	74 (92%)						
*New Tasks*									
*N*	75	72	75						
Duration	190.71 (137.93)	203.38 (161.83)	234.93 (173.97)	**1.71** **[1.08, 2.70]**	**2.30**	**.021**	1.45 [0.91, 2.30]	1.58	.114
Distance	159.68 (112.77)	166.92 (132.43)	206.05 (138.04)	**2.03** **[1.26, 3.28]**	**2.91**	**.004**	**1.72** **[1.05, 2.80]**	**2.18**	**.030**
Excluded^c^	4 (5.3%)	1 (1.4%)	11 (14.7%)						
Finished	72 (96%)	65 (90%)	68 (91%)						

*Notes*: CI = confidence interval; *SD* = standard deviation; VR = virtual reality. Data were analyzed at the task level. *p*-Values and CIs were reported with no adjustment made for multiple comparisons. Significant effects are shown in bold.

^a^Mean (*SD*) for continuous measures and *N* (%) for categorical measures.

^b^Estimated hazard ratio (95% CI). The “hazard” in this study was finishing the task, and hence desirable.

^c^Distances of trajectories with incorrect start/end points were calculated, and models fitted without imputation or missing data.

Demographic characteristics of randomized participants were similar across all groups (Table 2). The average age of the sample was 71.31 (standard deviation = 7.68); 38 (78%) reported as Female, 11 (22%) reported as Male, and none reported as Other.

**Table 2.** Demographic, Baseline, and Feasibility Measures by Condition Group

Measures	VR (*n* = 17)	Video (*n* = 16)	Control (*n* = 16)	Overall (*N* = 49)	Difference^a^	
					*F*	*p* Value
Age	70.35 (8.03)	71.06 (6.44)	72.56 (8.69)	71.31 (7.68)	0.35	.711
Sex (female)	14 (82%)	10 (62%)	14 (88%)	38 (78%)		.266
Ethnicity^b^						.422
White	17 (100%)	15 (94%)	15 (94%)	47 (96%)		
Asian	0 (0%)	0 (0%)	1 (6%)	1 (2%)		
Other	0 (0%)	1 (6%)	0 (0%)	1 (2%)		
MoCA	26.29 (1.99)	26.56 (1.93)	26.31 (2.60)	26.39 (2.15)	0.08	.927
Sense of Direction	4.33 (1.05)	4.99 (1.01)	4.68 (1.02)	4.66 (1.04)	1.69	.196
Computer Proficiency	27.53 (2.20)	26.59 (4.08)	27.88 (1.88)	27.34 (2.86)	0.85	.433
Mobile Device Proficiency	33.88 (7.12)	31.38 (6.95)	32.59 (6.80)	32.64 (6.89)	0.54	.589
Motion Sickness	13.86 (16.71)	31.09 (29.44)	10.99 (17.99)	18.55 (23.33)	3.92	.027
Usability^c^	63.82 (14.55)	59.06 (15.30)	70.00 (15.00)	64.29 (15.30)	2.15	.128
SS: Self-Location	3.71 (0.94)	2.72 (1.54)	2.41 (1.18)	2.96 (1.34)	4.97	.011
SS: Possible Actions	2.99 (0.96)	2.33 (1.23)	2.33 (1.40)	2.56 (1.22)	1.65	.203

*Notes*: MoCA = Montreal Cognitive Assessment; SS = Spatial Presence; VR = virtual reality.

^a^Difference between groups. *F*-test (2, 46) for continuous variables; Fisher’s exact test for categorical variables.

^b^No participants reported as “Black or African American,” “American Indian or Alaska Native,” or “Native Hawaiian or Pacific Islander.”

^c^Measured in all groups for intervention similarity.

### Primary Outcomes: Wayfinding Performance

**Figure 2.**
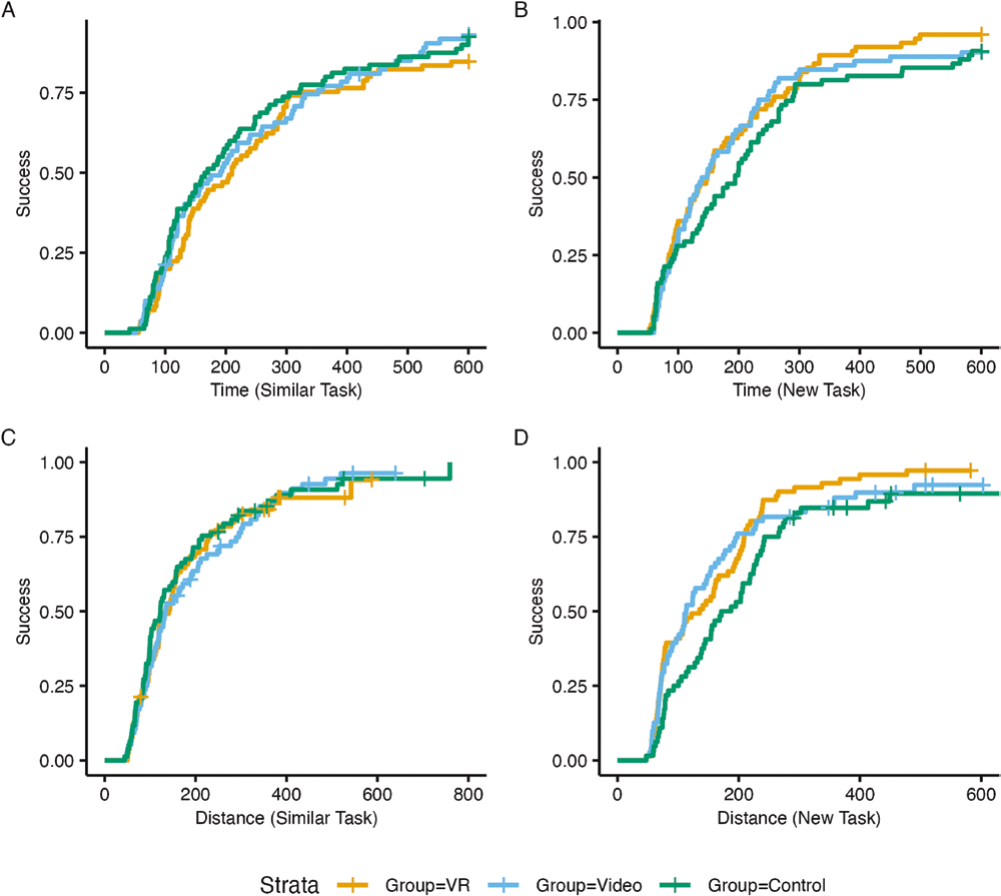
Wayfinding task success rates graphed against time spent on the tasks (**A** and **B**) and distance traveled during the tasks (**C** and **D**).

Wayfinding performance per group is summarized in Table 3 and illustrated in Figure 2.

In regard to the similar wayfinding tasks (those that were directly modeled in the training), there was no significant difference of the VR or Video training groups against the Control in terms of task Duration. However, the Video group performed significantly worse than the Control in terms of Distance Traveled (hazard ration [HR] = 0.68, 95% CI: [0.46, 1.00], *z* = −1.97, *p* = .049).

In regard to the new wayfinding tasks in the same building, the VR-trained group showed significant improvements over the Control in terms of both task Duration (HR = 1.71, 95% CI: [1.08, 2.70], *z* = 2.30, *p* = .021) and Distance Traveled (HR = 2.03, 95% CI: [1.26, 3.28], *z* = 2.91, *p* = .004). The Video-trained group also showed significant improvements over the Control in terms of Distance Traveled (HR = 1.72, 95% CI: [1.05, 2.80], *z* = 2.18, *p* = .030).

In sum, the training interventions did not seem to help when participants repeated the same tasks shown in the trainings, but these interventions did have an effect when participants completed new tasks in the same building. This could indicate a spatial learning or familiarization effect.

### Secondary Outcome Measures

The outcomes for wayfinding experience and spatial learning measures are summarized in Table 4.

**Table 4.** Effects of VR Training and Video Training on Secondary Outcomes

Measures	VR^a^	Video^a^	Control^a^	VR vs Control^b^	*t* (df)	*p* Value	Video vs Control^b^	*t* (df)	*p* Value
*Similar Tasks*									
*N*	85	80	80						
Spatial Anxiety	1.18 (0.26)	1.23 (0.41)	1.15 (0.24)	0.01 [−0.18, 0.21]	0.14 (45.0)	.889	0.09 [−0.10, 0.29]	0.97 (45.0)	.338
Workload	2.92 (1.16)	2.70 (1.21)	2.17 (1.16)	**0.71** **[0.13, 1.28]**	**2.47** **(45.0)**	**.018**	0.57 [−0.01, 1.16]	1.97 (45.0)	.056
Pointing Error	83.93 (47.67)	92.44 (46.43)	78.60 (55.74)	5.86 [−9.03, 20.75]	0.79 (45.0)	.432	13.36 [−1.72, 28.44]	1.78 (45.0)	.081
Distance Error	0.87 (0.65)	0.61 (0.49)	0.95 (0.98)	−0.11 [−0.50, 0.27]	−0.59 (45.0)	.556	−0.30 [−0.70, 0.09]	−1.57 (45.0)	.124
*New Tasks*									
*N*	75	72	75						
Spatial Anxiety	1.07 (0.16)	1.08 (0.19)	1.16 (0.31)	−0.09 [−0.25, 0.06]	−1.19 (40.8)	.242	−0.45 [−0.20, 0.11]	−0.59 (41.1)	.559
Workload	2.21 (1.04)	2.10 (1.14)	1.95 (1.06)	0.22 [−0.29, 0.73]	0.22 (40.6)	.395	0.20 [−0.30, 0.71]	0.20 (41.2)	.424
vPointing Error	69.45 (44.15)	71.65 (47.55)	67.83 (47.96)	3.88 [−15.37, 23.13]	0.41 (40.4)	.686	1.61 [−17.61, 20.83]	0.17 (41.1)	.866
Distance Error	0.97 (0.91)	0.92 (0.79)	1.08 (1.04)	−0.12 [−0.36, 0.13]	−0.96 (40.2)	.344	−0.14 [−0.38, 0.11]	−1.15 (41.0)	.258

*Notes*: CI = confidence interval; *SD* = standard deviation; VR = virtual reality. Data were analyzed at task level. *p*-Values and CIs were reported with no adjustment made for multiple comparisons. Significant effects are shown in bold.

^a^Mean (*SD*).

^b^Estimated mean difference (95% CI).

For most of these measures no significant differences were found in the VR group versus Control, or in the Video group versus Control. The sole significant difference was that Cognitive Workload was higher in the VR group than in the Control group (mean difference 0.71, 95% CI: [0.13, 1.28], *t*(45) = 2.47, *p* = .018). Workload did not significantly differ between the Video group and the Control group.

### Feasibility Measures

As noted in “Statistical Analysis”, participant-level user experience measures had limited statistical power in our study. The full outcomes for these measures are presented in Table 2. Most of the variables showed no significant differences between the intervention groups; however, perceptions of Self Location in the viewed material were significantly higher in the VR group than in the Control group, and Motion Sickness was significantly higher in the Video group than in the Control group.

## Discussion

In this study, we examined the effects of a VR wayfinding training intervention on subsequent real-world wayfinding performance, spatial learning, and wayfinding experience in an older adult population. The study moved beyond the common lab settings and evaluated the intervention effects in a real and functional building.

In terms of feasibility, none of the participants reported any major adverse effects. Reported levels of motion sickness in the VR training were low, in fact lower than that of the Video training group. Participants reported moderate–good usability ratings in respect to the VR environment (Bangor et al., 2009) and a high sense of Spatial Presence (especially on the Self Location subscale), which reflects the sensation of being within the virtual environment (Böcking et al., 2004). The VR training did exhibit a downside in that participants perceived cognitive workload as significantly higher in VR compared to the Control.

We did not find a significant wayfinding performance affect for VR training versus the Control group during the first set of real-world tasks, which were closely similar to those encountered during the training. However, in the second set of new tasks, the VR group significantly outperformed the Control. This somewhat incongruous and partial effect of VR training on the wayfinding performance is similar to previous comparisons of VR and 2D wayfinding training, where VR was suggested to have “some advantage” over 2D training but without significant difference between conditions (Hejtmanek et al., 2020). The impact of the VR training on wayfinding performance compared to the Control in our study also had effect sizes roughly similar to those previously found in cognitive training programs (Kelly et al., 2014).

Of particular note, however, is that a *delayed* benefit of VR training was reported by Verghote et al. (2019), who found that a wayfinding performance difference between 2D and 3D training conditions was present in the second half, but not the first half, of their study. This is similar to our own results, in which benefits were not found in the first set of similar tasks but were found in the subsequent set of new tasks. In other words, these findings for the new tasks might be related to repeated exposure, comprising both the VR training plus the similar tasks. It is possible that the VR training condition may have promoted more spatial learning across these multiple exposures (Meade et al., 2019; Parong et al., 2020), similar to what had been found for action video games (Green & Bavelier, 2012). We suggest that the higher sense of Spatial Presence in the VR may have fostered engagement and increased attentiveness to the environment (Kalantari et al., 2023; Pasqualotto et al., 2023). This interpretation is also supported by the significantly higher cognitive loads that participants reported for the VR condition relative to the Control.

Despite the difference in wayfinding performance, we did not find any differences between groups in the spatial learning measures (pointing task and distance estimation task). During the experiment, when completing these measures, some of the participants remarked that while they could easily lead us back to the task’s point of origin, they were less confident about their ability to point in its direction or estimate the straight-line distance. We note that an early study found that student nurses who spent 2 years in a hospital were able to find various rooms/locations but could not draw, or even understand, the floorplan of the building; these participants also had poor pointing and distance estimation outcomes, although they were highly familiar with the environment (Moeser, 1988). Later studies further supported the importance of sequential and first-person representations of space and corresponding route- and landmark-based wayfinding strategy (Iglói et al., 2009, 2010). It seems likely that these common methods of measuring spatial learning actually assess participants’ capability to form cognitive maps resembling floorplans, whereas many aspects of navigational knowledge, especially those utilized by older adults, do not rely on such maps, perhaps using landmark-based or turn-by-turn schemas instead (Colombo et al., 2017; Epstein et al., 2017; Kitchin, 1994).

It was rather unexpected to find that the highest motion sickness was in the Video group (Table 2), because such sickness is more commonly associated with VR. We speculate that this may be due to the specific production aspects and pace of movement through the building in the training video, especially because the Control video was not associated with a similar Motion Sickness outcome. The lack of participant control over motion in the training video (compared to the VR condition) may have contributed to this motion sickness, as the camera movements in the video were unexpected and not linked to active participant input. Revising the video production could likely improve this motion-sickness outcome, and possibly even improve the overall effectiveness of the Video training condition, though such conclusions are speculative. Overall, our results suggest that video training for a specific environment could potentially be an effective way to enhance wayfinding, but its impacts are likely to be weaker than VR training.

### Limitations and Future Studies

Our convenience sample was not entirely representative of the overall older adult population; it skewed heavily toward female and white participants, with high levels of Computer Proficiency and Mobile Device Proficiency compared to previous studies (Boot et al., 2015; Roque & Boot, 2018). Future researchers could benefit from applying a more systematic sampling method to improve participant diversity. Furthermore, whereas self-reported Sense of Direction among our participants was similar to older adults recruited in other studies (Gandhi et al., 2021), future researchers could benefit from targeted recruiting of more participants with a lower Sense of Direction, and consider subgroup analysis based on navigational abilities to examine the intervention effect on those who might benefit the most from such interventions (Verghote et al., 2019). Future studies may also benefit from recruiting participants with a higher age threshold (e.g., minimum age of 65 years, rather than the minimum of 58 years used in the current study) to better represent the population that is most suspectable to cognitive decline and could potentially benefit most from the intervention.

It is notable that because the primary outcome studied in the current study was wayfinding performance, our user-experience metrics were analyzed with reduced statistical power because this data was collected at the participant level rather than at the trial/task level. This could result in an increased risk of type II error (false negatives) when considering differences in user experiences between the VR, Video, and Control conditions. Furthermore, as discussed in “Procedure” section previously, all participants were asked to complete a training session about use of the VR equipment prior to the various interventions (VR, Video, or Control). This approach was intended to minimize differences in the procedure between groups; however, it may have affected our user-experience data, again potentially leading to risk of type II error as it reduced the differences in the intervention regimens. Future studies on user experience may consider separating the VR control training and the intervention with a break (one day or longer) to minimize such risk.

Future studies may also benefit from examining different choices of design elements in the VR intervention. Some notable considerations in this regard are the control interface and means of movement in the simulation (locomotion vs teleportation) (Jerald, 2015), the (adaptive) difficulty of navigational training tasks, and the variety of environments encountered in training (Kelly et al., 2014; Stine-Morrow et al., 2024). VR training platforms with more natural movement and a greater variety of challenges might produce greater training outcomes beyond the target building. Future studies may also explore the dose–response relationships (number of sessions and length of each session) and directly compare outcomes between different digital training platforms and environmental designs to better determine the best choices of activities and features.

Although our results provided evidence supporting the VR training to be effective, Video training was also found to be possibly beneficial with weaker evidence. The cost of intervention should also be taken into consideration: despite the reported motion sickness and possible lower efficacy, video intervention is more likely to be affordable and approachable considering its lower learning- and financial cost. Future studies should keep exploring the potential of 2D screen-based trainings for wayfinding, both in the form of passive video tours and interactive training programs with different means of movement.

Finally, in this study, we examined only the short-term effect of a single-session training on a very direct target: wayfinding performance in a simulated environment. Although the current study did not prespecify nor include cognitive functions as outcomes, effects on cognitive abilities related to wayfinding could be examined in a longitudinal study following regular exposure to training sessions over time.

In conclusion, this study indicates that VR wayfinding training is feasible with older adults and provides some evidence that this type of training can effectively improve wayfinding performance in older adults. Although participants in the VR condition reported higher workload, there were no reported adverse effects. The effects of video training on performance were not found to be significantly different from control and were estimated to be slightly worse than the VR condition with a very small effect size. Future studies should include larger samples and include participants who have challenges with wayfinding, explore different VR training design features, and examine the long-term effect of simulated wayfinding training.

## Supplementary Material

igae099_suppl_Supplementary_Table_S1_Figures_S1-S2

## Data Availability

De-identified dataset are available at OSF (https://osf.io/63t5z/). The study was not preregistered.
