# Supplementary material for: Assessing the Feasibility and Efficacy of Virtual Reality Navigational Training for Older Adults
Source: Innov Aging. 2024 Dec 12;9(1):igae099. doi: 10.1093/geroni/igae099 (PMC11705671; doi:10.1093/geroni/igae099)

***Innovation in Aging* Supplementary Material: Xu et al. Assessing the Feasibility, and Efficacy of Virtual Reality Navigational Training for Older Adults.**

**Supplementary Figure 1.** Overview of the experiment procedure.


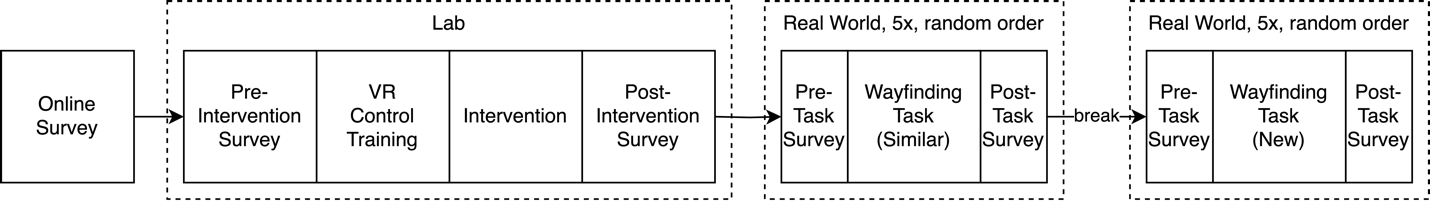


**Supplementary Table 1.** Summary of Wayfinding Tasks.

| **Task Number** | **Start Location** | **End Location** | **Change in Floor Level** |
| --- | --- | --- | --- |
| 1 | T70 | Café | No (Single level) |
| 2 | Café | 1250 | Yes (Multi-Level) |
| 3 | 1250 | 1106 | No (Single level) |
| 4 | 1106 | G151 | Yes (Multi-Level) |
| 5 | G151 | T70 | Yes (Multi-Level) |
| 6 | G333 | 1300 | Yes (Multi-Level) |
| 7 | 1300 | 1210 | No (Single level) |
| 8 | 1210 | 1429 | No (Single level) |
| 9 | 1429 | T115 | Yes (Multi-Level) |
| 10 | T115 | G333 | Yes (Multi-Level) |

*Note:* Tasks 1–5 were closely similar to the intervention training; Tasks 6–10 took place in a part of the building that was not covered in the training.

**Supplementary Figure 2.** Floorplan view of the wayfinding tasks.


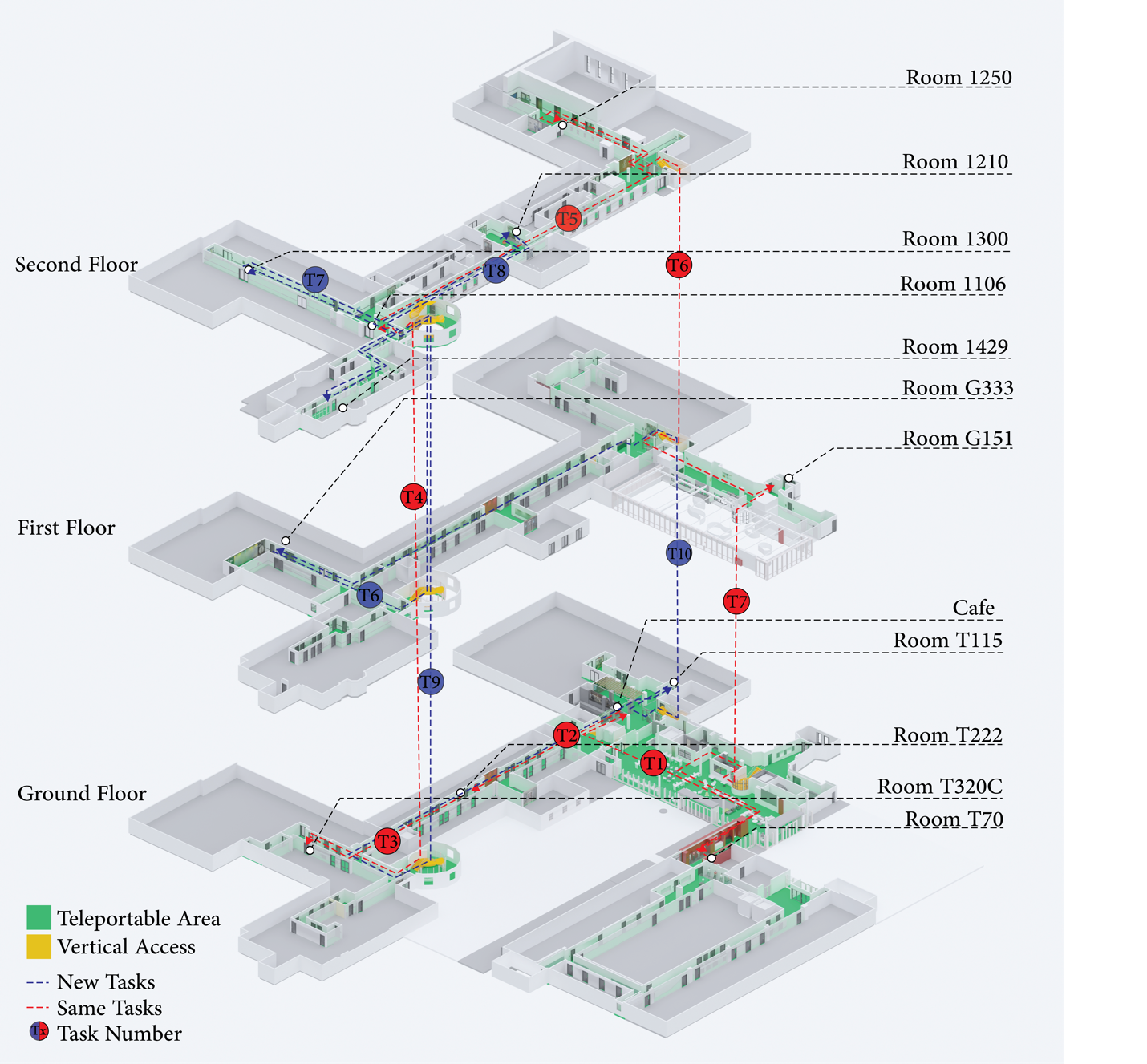

Supplement: igae099_suppl_Supplementary_Table_S1_Figures_S1-S2 [file igae099_suppl_supplementary_table_s1_figures_s1-s2.docx]
